# Amelioration of patients with chronic spontaneous urticaria in treatment with vitamin D supplement

**DOI:** 10.1186/s12948-017-0078-z

**Published:** 2017-12-22

**Authors:** Nazila Ariaee, Shima Zarei, Mojgan Mohamadi, Farahzad Jabbari

**Affiliations:** Allergy Research Center, Ghaem Hospital, Mashhad University of Medical Sciences, Shariati Square, Mashhad, Iran

**Keywords:** Spontaneous chronic urticaria, Vitamin D, T reg

## Abstract

**Background:**

Spontaneous urticaria is a common allergic skin condition affecting 0.5–1% of individuals and may burden on health care expenditure or may be associated with remarkable morbidity.

**Aim:**

In this study, we measured the effect of vitamin D supplementation in patients with a diagnosis of CSU. Furthermore, quality of life and cytokine changes were evaluated.

**Methods:**

The clinical trial was conducted on 20 patients with idiopathic chronic urticaria. Vitamin D was administered orally for 8 weeks and disease activity was measured pre- and post-treatment using USS and DLQI. On the other hand expressions of IL-17, IL-10, Foxp3, and TGF-β by Real-time RT-PCR were assessed.

**Results:**

USS questionnaire showed that severity of idiopathic urticaria after the intervention, which compared with the first day reached a significant 55% reduction. The DLQI quality of life questionnaire 2 months after treatment showed 55% improvement. Along with the significant improvement of clinical symptoms, use of vitamin D increase FOXP3 gene expression and downregulation of IL-10, TGF-B, and FOXP3, IL-17, but these changes were not statistically significant.

**Limitation:**

These might happen due to lack of enrolled population in the investigation.

**Conclusion:**

Vitamin D can be used along with standard medical care and it’s a safe and cost-effective method for the treatment of chronic urticaria with deficiency of vitamin D.

## Background

Chronic spontaneous urticaria (CSU) is a common allergic skin condition affecting 0.5–1% of individuals and may burden on health care expenditure or may be associated with remarkable morbidity [1]. It is characterized by urticarial itchy-wheals occurring almost daily and lasting more than 6 weeks, a remarkable proportion of the urticaria patients experience this condition for 10 years [2]. According to various recommendations; the diagnosis of the disease depends on clinical parameters, and therapeutic management still remains almost unclear. Generally, more severe urticaria symptoms are, more difficult to treat [3].

Many of the patients with CSU, approximately 35–40% have circulating autoantibodies against the immunoglobin E (IgE) or against high-affinity receptor for IgE (FcεRI). These patients are considered to have CSU. Some studies suggest of T lymphocyte and its related subpopulations including T helper 1 (Th1), T helper 2 (Th2), T regulatory (Treg) cells particular roles in the onset and maintenance of chronic inflammatory responses in CSU [4].

There is a vivid association with the T cell subpopulation, especially Tregs and Th 17, of such patients with autoimmunity [5]. H1-antihistamines (second-generation) are recommended as first-line therapy [6]; the doses of antihistamines and choosing the most appropriate antihistamine or other associated drugs including anti-leukotrienes, immunomodulatory, and immunosuppressives are not specified [7].

A role for vitamin D (Vit D) in allergic diseases increased attention in recent decades when the causative role for it in the treatment of the immune-mediated issue, including some autoimmune diseases, cancers, transplant rejection, had been proved [8]. Furthermore, a large number of investigations indicated vitamin D deficiency had linked with asthma, dermatitis or even allergic rhinitis [9]. It also displayed considerable functions in anti-inflammatory or immunoregulatory diseases [10].

In this study, we tried to evaluate Vit D level in sera of chronic spontaneous urticaria patients and had a treatment with Vit D supplement to assess the outcome in the alleviation of the severity of urticaria and life quality of enrolled patients. Moreover, the Tregs specific cytokines such as Transforming growth factor β (TGF-β), Interleukin 10 (IL-10), Interleukin 17 (IL-17), Forkhead box P3 (FOXP3) concentrations were compared before and after the intervention to evaluate the possible role of Vit D in Treg function and its relation to chronic idiopathic urticaria.

## Methods

### Patients

Chronic urticaria patients who referred to Allergy Clinic, Ghaem hospital (Mashhad University of Medical Science, Mashhad, Iran) from March 2014 to March 2015, were enrolled. The patients were visited separately by two different allergists and entirely fulfilled the criteria of idiopathic chronic urticaria [13].

All the patients should have a history of involving with urticaria at least for 6 weeks of and 4 days a week with Vit D concentration lower than 10 ng/ml were selected to study. In addition, the negative allergy skin prick test for common local aeroallergens [11], and foods allergies were checked patients with allergic rhinitis were excluded from the study. After that their CH50 and IgE level were evaluated, patients with normal CH5 and total IgE level (less than 150 IU per ml) and CH50 were selected for the study. Patients were also evaluated by autologous test to find if they have active autoantibodies, to eliminate patients with the active autoantibodies meanwhile, patients with auto immune diseases were also excluded. In addition, all patients were checked for normal hematological indices including Complete blood count, erythrocyte sedimentation rate. While they were checked for normal biochemical factors such as blood urea nitrogen, creatinine, and leaver functional tests. They were also checked for normal C-reactive protein, Antinuclear Antibody, beside that negative Hepatitis B and *Helicobacter pylori* antigens were considered. Patients with Vit D concentration higher than 30 ng per ml were also excluded.

Patients had administrated with the highest dose of antihistamines for 3 months before adding Vit D supplement to their treatments strategy. Antihistamines were selected distinctly from patients with urticaria usually had demonstrated different responses to the various treatments so the most appropriate one had chosen for every individual. After these 3 months of treatment, our intervention was initiated, and oral Vit D was added to their conventional treatment. They have administrated a pearl of 50,000 unit every week during 8 weeks. The effects of intervention with Vit D compared with before intervention, so there was no inquiry of the control group.

Firstly, quality of life and urticaria severity were evaluated, by filling Dermatology Life Quality Index (DLQI) and Urticaria Severity Score (USS) questioners with a range of 0–30 and 0–56, respectively. Two mounts after addition of Vit D supplement to the conventional treatment as previously talked, the questioners filled again to evaluate urticaria severity and patients’ quality of life and comparing with previous one.

### Sample collection and gene expression

To evaluate cytokine changes, before and after intervention 8 cc brachial blood was obtained from patients and after isolating peripheral blood mononuclear cell (PBMCs), the cells were cultured in RPMI (Fermentas, Canada). The PBMCs were harvested after stimulating by Phytohaemagglutinin (PHA) when 6 h lasting. Afterward, RNA extracted and cDNA was synthesized (Fermentas, Canada). cDNA quality checked by PCR with GAPDH universal primers. The process followed by SYBR Green real-time PCR of interested genes including IL-10, IL-17, TGF-β, FOXP3, β2ϻ gene and its universal primers was used as internal control the sequence of primers can be seen in Table 1. All the PCR products were sequenced to clarify the accuracy of Real-time PCR amplification in Bioneer Company, South Korea.

**Table 1:** Designed primers for genes of interest

Name	Accession number	Product length (bp)	Sequence (5′ → 3′)
IL-10	NM_000572.2	111	Forward: TTGCTGGAGGACTTTAAGGGT Reverse: CTTGATGTCTGGGTCTTGGTT
IL-17	NM_000619.2	142	Forward: GTCAACCTGAACATCCATAACCG Reverse: ACTTTGCCTCCCAGATCACAG
FOXP3	NM_000589.2	95	Forward: ACTACTTCAAGTTCCACAACAGC Reverse: GAGTGTCCGCTGCTTCTCTG
TGFβ	NM_000660.5	192	Forward: GCAAGTGGACATCAACGGG Reverse: CGCACGCAGCAGTTCTTCTC

### Statistical analysis

Data analysis was carried out using the SPSS software (version 16, USA). To analyze the normal distribution of cytokines in both groups, the Kolmogorov–Smirnov test was applied. As a result of the normal distribution of data in both groups, a paired t test was applied to compare the averages before and after treatment. In the case of abnormal distribution, Mann–Whitney test and other nonparametric tests were applied. Differences were considered to be significant when p < 0.05.

## Results

### Demography

In this investigation, 20 patients with urticaria were visited in Allergy clinic during the year of study. The mean age of the patients was (Mean ± SD) 35.6 ± 13.1 while the youngest patient was 14 and the oldest one was 57 years old. 11 (55%) of them were females and other 9 (45%) were males. The average time that patients involved with urticaria was 22.1 ± 7.6 months.

### Clinical effects

According to DLQI, the quality of life of enrolled patients was changed after treatment with Vit D supplements and their quality of life was significantly increased. In this case, according to the parameters of DLQI, and based on patients’ answers, patients were subdivided into 4 groups including without effect, low, moderate and intensive effects. Vit D was the dramatically inhibited impact of chronic urticaria on the quality of lives in all 4 groups. The details of DLQI outcomes can be seen in Table 2.

**Table 2:** Quality of life Index before and after treatment with Vit D

	DLQI in CSU	N	Mean ± SD	Mean ± SD
Before treatment with Vit D	No effect	2	1	10.8 ± 1.6
	Low	9	6 ± 2	
	Moderate	7	16.1 ± 3.1	
	Severe	2	23.5 ± 0.7	
After treatment with Vit D	No effect	5	0.6 ± 0.54	0.9 ± 4.8
	Low	13	5 ± 2.7	
	Moderate	2	13.5 ± 3.6	
	Severe	0	0	

The severity of idiopathic urticaria in conducted patients after treatment with Vit D was statistically decreased. According to USS parameters enrolled patients were subdivided into 4 groups including very low, low, moderate, and severe. The amount of the abatement was 55% after the intervention. The details of the USS outcomes in both stages including before and after treatment with Vit D can be vividly seen in Table 3.

**Table 3:** Severity of urticaria according to USS questionnaire parameter before and after treatment with Vit D

	Urticaria severity score	N	Mean ± SD	Mean ± SD
Before treatment whit Vit D	Very low	0	0	23.5 ± 13.9
	Low	9	10.3 ± 3.1	
	Moderate	3	23.3 ± 2.5	
	Severe	8	38.4 ± 6.1	
After treatment whit Vit D	Very low	0	0	11.2 ± 9.6
	Low	13	5.1 ± 2.5	
	moderate	6	19.5 ± 2.6	
	Severe	1	39	

### Cytokines assay

The results of SYBR Green Real-time PCR for TGFβ gene transcript apparently indicated that TGFβ expression was dropped out after treatment with Vit D. The outgrowth decline was not statistically significant, the mean ± SD of the TGFβ was1.42 ± 0.43 at the first, and after the intervention, it decreased only to 0.98 ± 0.2.

The result of the assessment of IL-10 expression before and after the intervention indicated that the concentration of this cytokine was 2.7 ± 0.7 and during the treatment it decreased and achieved to 1.4 ± 0.3, the average of IL-10 was dropped out but not significantly according to paired t test in p < 0.05.

Evaluating IL-17 before and after treatment with Vit D in chronic idiopathic urticaria showed that the amount of IL-17 expression was dropped out from 2.4 ± 0.6 to 1.1 ± 0.6. Although the decline was reached to half of primer amount, this decline was not statistically significant.

Our data showed that FOXP3 was increased after treatment with Vit D and raised from 0.65 ± 0.2 to 0.77 ± 0.1 but the rise was not also significant.

## Discussion

We undertook this study based on the hypothesis that vitamins and minerals effect some allergic issues [12] and the reports that proved that Iranian population had a profound deficiency of Vit D [13]. Meanwhile, some other studies reported the variety of gene expression and their fluctuation, and cytokine changes involved in urticaria, the furthermore presence of different T cell subsets in active lesions of patients with CSU were observed [14]. So we tried to uncover associations of Vit D with severity CSU and the possible role of T reg and Th17 related cytokines.

Previously, some authors reported symptom alleviation of urticaria after administration of vitamin D supplementation [15]. Our data also support this theory in different ways regarding improving health outcome, quality of life, and severity of the disease in a patient with CSU. In comparison with similar works that evaluate patients with urticaria, we tried to have strict inclusion criteria to have evaluation only for CSU; since it is more challenging to find a specific diagnostic strategy [16].

In this study, we revealed that FOXP3, a clinical determinant of Treg, increased in CSU patients after treating with Vit D supplement. The data were aligned with other studies focused on the phenotype or functional analysis of Tregs, they had revealed Treg which isolated from patients with autoimmune disorder reduced regulatory function comparing with healthy individual Tregs [17]. Treg devoted to aiding immune tolerance, hence their expansion is necessary for the resolution of inflammation and in preventing tissue damage or autoimmunity [18]. Tregs are usually characterized by the expression of FOXP3, which is the main transcription factor and also a master regulator of the immune suppressive activity of Treg [19]. It may be concluded that increase percentage of FOXP3 contributes to reducing the autoimmune pathogenic process of CSU. Although in our study, there was no significant rise in the concentration of FOXP3 in CSU patients before and after intervention in p > 0.05, the results were consistent with others who exhibited an increasing number of Tregs and FOXP3 expression while alleviating CSU symptoms.

Our investigation revealed that IL-17 had decreased after treatment with Vit D supplement in patients with CSU. In several skin diseases were proved previously that Treg and Th17 immune cells have to oppose immunomodulatory act in the inflammatory process [20], so we expected to have decreased in IL-17 while we had an increase in FOXP3. The opposing functions of Treg and Th17 cells have led many studies to suggest that unbalanced Th17/Treg outcome may be involved in the pathogenesis of some chronic inflammatory diseases [21]. These findings were confirmed by our experiments in which the constituent ratio of IL-17 cytokines significantly decreased. The imbalance of Th17/Treg cell subsets has been proposed to involve in CSU pathogenesis and functional role in ameliorating patients with CSU [22, 23]. It speculated that inflammation can be subsided by the balance of Th17/Treg cell populations [24]. However, the mechanism still remains unclear. Further study may determine delicately how each cytokine interplays to exhibit the disease severity [25].

In evaluating IL-10 concentration, it was found that it had the reduction after treatment with Vit D in patients with CSU. Some studies have been previously shown levels of IL-10 significantly increased in the sera or cutaneous samples of patients with CSU, compared with the healthy population which is consistent with our data [26]. By contrast, some other studies discovered patients with CSU had lower serum levels of IL-10 compared with the healthy population [27].

IL-10 is an anti-inflammatory cytokine that secreted by some immune cells like T lymphocytes and macrophages [28]. It has inhibitory effects on mast cells and also controls proinflammatory cytokine release or their function [29]. It is not yet understood what is the specific role of IL-10 in urticaria, and still remain unclear this increase/decrease in IL-10 is a consequence of onset of CSU or compensatory response [26, 28].

Same as happened for IL-10, we had experience attenuation of TGF-B after treatment with Vit D in Patients with CSU. TGF-β has been demonstrated to have the ability to promote apoptosis of mast cells. Meanwhile, it can suppress the expression of both of the FcεRI and IgE on mast cell surface [29]. Furthermore, it interferes with mediator release from mast cells so it may play an important role in homeostasis of mast cells [30]. Some studies illustrated that TGF-β can be a marker of mast cell degranulation and it usually rises during the active stage of urticaria. However; Anti-histamines help relieves the symptoms in some patients, some other patients do not respond sufficiently to this treatment due to the complexity of CSU pathogenesis [2]. such as TGF-β1 and IL-10 as their immunoregulatory function inhibit both Th1 and Th2 cells [31], so we expected to have dropped out of this two cytokine by amelioration of patients with CSU after treatment with Vit D.

CSU is a heterogeneous group of disorders, and it is unclear if this proportion of subjects [20] with CSU can truly represent all aspect of this disorder, so limitations of this study include the small number of subjects and lack of information about Th2 cytokines regarding cell specificity associated with altered gene expression. The single time point sampled during treatment might be beneficial to prove changing mentioned cytokines during the intervention. Furthermore, we did not have any placebo group so we may have some placebo effects.

## Conclusion

In conclusion, our findings showed that Vit D supplement had a profound impact on CSU severity and patients’ quality of life. Since Vit D is a cost-effective, profitable, and safe alternative that can be added to any therapeutical strategy in the management of the disease. On the other hand, due to its tremendous effects, it may apply in order to reduce other treatments like corticosteroids and immune suppressive. Moreover, the adverse side effects of conventional treatment can be avoided in this way.
